# Needs assessment of school and community physical activity opportunities in rural West Virginia: the McDowell CHOICES planning effort

**DOI:** 10.1186/s12889-015-1702-9

**Published:** 2015-04-03

**Authors:** Alfgeir L Kristjansson, Eloise Elliott, Sean Bulger, Emily Jones, Andrea R Taliaferro, William Neal

**Affiliations:** School of Public Health, West Virginia University, Robert C. Byrd Health Sciences Center, 1 Medical Drive, Morgantown, WV 26505 USA; College of Physical Activity and Sport Sciences, West Virginia University, Morgantown, WV 26506 USA; School of Medicine, West Virginia University, Robert C. Byrd Health Sciences Center, Morgantown, WV 26506 USA

**Keywords:** Physical activity, Public schools, Community needs assessment, Rural health

## Abstract

**Background:**

McDowell CHOICES (Coordinated Health Opportunities Involving Communities, Environments, and Schools) Project is a county wide endeavor aimed at increasing opportunities for physical activity (PA) in McDowell County, West Virginia (WV). A comprehensive needs-assessment laid the foundation of the project.

**Methods:**

During the 6 month needs assessment, multiple sources of data were collected in two Town Hall Meetings (n = 80); a student online PA interest survey (n = 465); a PA and nutrition survey among 5^th^ (10–11 years) and 8^th^ graders (13–14 years) with questions adapted from the CDC’s Youth Risk Behavior Surveillance Survey (n = 442, response rate = 82.2%); six semi-structured school and community focus groups (n = 44); school site visits (n = 11); and BMI screening (n = 550, response rate = 69.7%).

**Results:**

One third of children in McDowell County meet the national PA minimum of 60 minutes daily. At least 40% of 5^th^ and 8^th^ graders engage in electronic screen activity for 3 hours or more every day. The prevalence of obesity in 5^th^ graders is higher in McDowell County than the rest of WV (~55% vs. 47% respectively). SWOT analyses of focus group data suggest an overall interest in PA but also highlight a need for increase in structured PA opportunities. Focus group data also suggested that a central communication (e.g. internet-based) platform would be beneficial to advertise and boost participation both in current and future programs. Schools were commonly mentioned as potential facilities for public PA participation throughout the county, both with regards to access and convenience. School site visits suggest that schools need more equipment and resources for before, during, and after school programs.

**Conclusions:**

An overwhelming majority of participants in the McDowell CHOICES needs assessment were interested to participate in more PA programs throughout the county as well as to improve opportunities for the provision of such programs. Public schools were widely recognized as the hub of the communities and provide the best venue for PA promotion for both students and adult citizens, and can potentially serve as a platform for change in rural communities such as McDowell County.

**Electronic supplementary material:**

The online version of this article (doi:10.1186/s12889-015-1702-9) contains supplementary material, which is available to authorized users.

## Background

Engaging in regular physical activity (PA) has long been associated with positive health outcomes, including prevention and treatment of obesity [1-5]. As childhood obesity continues to be a public health concern, daily PA participation is critical for children’s health, and should be considered in all school and community environments [6-9]. A number of factors have been found to influence the PA levels of school-aged youth on a consistent basis: those include (a) demographic and biological; (b) psychological, cognitive, and emotional; (c) behavioral attributes and skills; (d) social and cultural; and (e) factors in the build environment [10]. The development of effective physical activity interventions for children and adolescents is in part dependent on the main modifiable factors such as physical activity preferences, readiness for change, perceived competence, social support and direct help, program/facility access, time spent outside, and access to sport and physical activity opportunities in the community.

Recent studies have also positively associated PA among children and youth with improved academic learning abilities and higher scholastic achievement [11-13]. The CDC [1] has identified that the creation and/or enhancement of access to PA spaces represents an effective and sustainable approach to improving public health. Improving conditions and opportunities for PA in communities is therefore an important objective for future health and well-being. The purpose of this study was to gain insight into school and community factors that influence PA opportunities for children and their families in McDowell County, WV, and to understand improvements needed to promote both structured and unstructured PA participation.

### West Virginia and McDowell county

National surveillance of health indicators reveal that WV consistently ranks as one of the lowest in most health indices and is a forerunner in the obesity epidemic [14,15]. WV is ranked first nationally for the prevalence of heart attack and coronary heart disease among adults, second for diabetes [16], and third for obesity [17]. Statewide surveillance data has shown that McDowell County consistently has a high prevalence of chronic disease and health risks. According to the Robert Wood Johnson’s 2014 County Health Rankings, McDowell County ranked last (55th) in WV relative health outcomes (how healthy a county is) and health factors (what influences the county’s health) [18]. The premature death rate of McDowell County citizens (17,644 per 100,000 population) was over three times the national benchmark (5,466), and two-fold that of WV (9,351), placing McDowell last among the 55 counties in the state. McDowell was ranked 53^rd^ in morbidity, a general measure of perceived quality of life, with a reported 35% of residents citing poor or fair health [18]. The proportion of sedentary adults in WV was also higher than the national average, with 27.6% of residents who reported no leisure exercise, as compared to 24.2% nationally [16]. McDowell County has reportedly been the most sedentary county in WV with 44% of adults reporting no leisure exercise and 36% being obese [16]. In addition, data from the 2013 CDC Youth Risk Behavior Surveillance System showed that 69% of high school children in WV were not physically active for the recommended minimum of 60 minutes per day during the 7 days prior to the survey which was akin to the national average at the time [19]. Furthermore, around 62% of WV high school students did not attend PE in an average week compared to about 52% nationwide [19].

McDowell County in WV is a rural county. Rural living is often times synonymous with poorer quality of life, including poor health, poverty, and lack of opportunities [14]. West Virginia is the second most rural state in the US, with about two-thirds of its population living in communities with a population less than 2500 [20]. In McDowell County, there are over 80 unincorporated communities, with a countywide population of only 22,113 [21]. Studies have shown that access to recreational facilities designed to engage in PA and transport options such as the presence of sidewalks and access to public transportation are critically important for the availability and opportunity to engage in PA [22,23]. However, rural areas are faced with some unique challenges, including overall isolation and scarcity of infrastructure, challenging road conditions, relatively long distances, lack of public transport, and often high rates of poverty [14,24]. The geography of WV, and in particular McDowell County, is rugged and mountainous, which increases time and distance to travel from one place to another. In McDowell County, there are numerous narrow and winding secondary roads, with no interstate roadways available. Poor weather conditions in the winter months add more time and barriers to travel. These rural environments often times influence a culture separated from other cultural groups and resources of those groups, therefore interventions become an important part of health initiatives.

Currently, the rural McDowell County ranks as one of the poorest counties in the Nation with a median household income of $22,154, well below the WV state average of $38,380. School-aged children make up 25.7% of the population, with 16.6% over the age of 65. McDowell County has one of the largest minority populations among WV counties (10.9%), with 9.3% black and 1.6% other ethnicities [18,21]. Related to education, the most recent high school graduation rate in McDowell County was only 59.6%, compared to the WV average of 81.9% [21]. Similarly, only 29% of residents report some college experience, compared to the national and state averages of 68% and 49%. These barriers call for a better understanding of the opportunities and challenges for PA in McDowell County and other rural communities, and targets of change that could be addressed.

### The current study

This study is guided by the principles of community organization [25] through the lenses of the social action model [26]. The social action model in community organizing is a task and process-oriented model where external expertise is provided to assist communities to both identify problems and set common goals to overcome them, as well as to develop strategies to mobilize existing resources to influence positive development. Three key concepts guided our work: issue selection, community capacity, and empowerment (based on [25], pp. 294):

1. *Issue selection* concerns the identification of winnable and specific targets of change that may contribute to building community strength. For the purpose of this needs assessment we begin our issue selection simply by asking whether increase in PA opportunities are likely to be of importance to the citizens of McDowell County.
2. *Community capacity* concerns characteristics that affect the community’s ability to identify, mobilize and address a given problem. In this respect community capacity addresses the question of whether citizens of McDowell County are able and willing to participate in identifying ways to improve conditions for PA opportunities, as well as plans to address hurdles in the way.
3. *Empowerment* is the process by which people gain mastery over their lives and their communities. For the purpose of this study empowerment addresses the belief and ability of community members in McDowell County to assume ownership over the issue of increasing PA opportunities as well as increase in power to facilitate desired changes.

In order to establish baseline knowledge of currently available resources and utilities of existing opportunities for PA in McDowell County, and consistent with the social action model of community organizing, a comprehensive needs assessment using both qualitative and quantitative methods was carried out. This inquiry included 1) two Town Hall Meetings with the general population, 2) two sets of focus groups with school personnel and other community members, 3) a PA interest survey among a selection of students from all middle and high schools, 4) a PA and nutrition survey among all eligible 5^th^ and 8^th^ grade students, and 5) school site visits to every school that included interviews, equipment inventories, and a facilities audits. Additionally, as part of the WV statewide screening provided by the WV Coronary Artery Risk Detection in Appalachian Communities Project (CARDIAC) the body mass index of all consenting children in 2^nd^, and 5^th^ grade was measured [27].

## Methods

### Participants and procedures

#### Town hall meetings

Two Town Hall Meetings (THM) were hosted in collaboration with McDowell County Schools. The meetings were open to all interested community members and took place in the two county high schools during February 2013. The primary purpose of these meetings was to provide an overview of Senate Bill 371 (Governor’s School System Collaborative Innovation Zone), to introduce the McDowell CHOICES program, and to solicit feedback related to interests and recommendations for enhancing PA. During the meeting interested attendants responded to a survey of 9 questions (N = 80). The data were collected using an audience response or clicker system which enabled the participants to respond to the facilitated-presented questions using a digital format. Participant responses were recorded and displayed in real-time which allowed for immediate feedback and dissemination of results in graph form. Results were projected to the audience to facilitate discussions related to the findings. Each meeting lasted 1 hour with about 20 minutes allocated to the survey and related discussions.

#### Physical activity interest survey

An online PA interest survey open to all interested students in grades 6 through 12 was conducted from May 20 – June 3, 2013. The survey included the names and visual illustrations of 35 items pertaining to physical activities and sports deemed of interest to students in the county, and further 11 items on attitudes and norms around PA opportunities and participation. Students were asked to rate each activity according to their interest in participating either during physical education class, after school, or outside of school. The rating scale used was a 4 point Likert scale. The rating scale descriptors were chosen based on positive results from discussions with middle/high school students and their teachers who used the scale in a previous study. Four hundred and sixty-six students from all five middle and high schools in McDowell County responded to the survey with 75% of participants being from grades 6–8.

#### Student PA and health-related behavior survey

As the main program evaluation component of the McDowell CHOICES program a PA survey was conducted with all 5^th^ (10–11 years) and 8^th^ grade students (13–14 years) in all 11 schools of McDowell County during May of 2013. In line with the Family Educational Rights and Privacy Act (FERPA) [28], the survey was carried out with permission from McDowell County Superintendent of schools to pre-enroll the name, gender, date of birth and WVEIS number of students in those grades into the data file. FERPA allows schools to disclose those records without parental consent to organizations conducting health related studies for or on behalf of the school. The survey included 30 items pertaining to PA and sedentary lifestyle, selected nutrition measures and questions concerning attitudes and ideas about PA that were adopted from the annual CDC’s Youth Risk Behavior Surveillance Survey [19]. Total number of respondents was 442 out of 533 registered students in those grades (Response rate: 82.2%, boys 51.6%).

#### Body mass index measurement

As part of the annual WV state-wide Coronary Artery Risk Detection in Appalachian Communities Project (CARDIAC) all consenting children in McDowell County attending grades 2, 5 and 8 were physically measured for height and weight during March of 2013 (n = 550, response rate: 69.7%). Using professional grade stadiometers and digital scales, BMI percentiles were then calculated by comparing each child’s BMI values using the EpiInfo age and gender-specific growth charts. Categorization of overweight (85–94.9th%), and obese (≥95th%) is based on official cutoff scores from the Centers for Disease Control and Prevention [29].

#### Focus groups

Two sets of three semi-structured focus groups were conducted using a semi-structured SWOT (strengths, weaknesses, opportunities, and threats) analysis framework to gain insight into communities’ unique SWOT with regards to PA. The first three (3) focus groups were carried out during February 28^th^ 2013 with 2 representatives from each of the 11 schools (n = 22, Females = 15). In addition, three (3) focus groups were conducted among adult community members in the city of Welch during March 1^st^ 2013 (n = 22, Females = 17). Participation was open to all interested citizens and was conducted after the conclusion of a monthly community meeting. Each focus group lasted 45–60 minutes. The group responses were recorded and transcribed by an external professional transcriber. Concept mapping and thematic coding was then employed to analyze the data that consisted of 132 transcribed single-space pages and just over 5 hours of audio material. Each aspect of the SWOT data collection was organized around four thematic areas; individuals, families, schools and the environment. The focus group results were summarized using the following three layer truncated protocol: 1) First, each transcript was thoroughly reviewed and summary files created for strengths, weaknesses, opportunities, and threats around the aforementioned four semi-structured thematic areas. During this review particular notations were made of recurring discussion points by participants. 2) The results from #1 were further summarized to a single page summary table for each SWOT group with particular emphasis on the most frequently mentioned issues. Additionally, 1–2 direct quotes from participants that highlighted each issue was copied and saved into the file. 3) Finally, the tabulated data from #2 was abbreviated into 1–2 paragraphs for each of the SWOT subsections and a single descriptive quote from participants selected to bring “live” to the data.

#### School site visits

School site visits were conducted in all 11 McDowell County schools during March 2013 to gain insight and understanding of the multidimensional nature of schools and examine the organizational structure and climate for PA with a special focus on Comprehensive School Physical Activity Programs (CSPAP). Each visit was comprised of 1) two 30–45 minute interviews – one with the school principal and one with the physical education (PE) teacher (total of 22 interviews), 2) collection of needs assessment inventories related to PE equipment, PE curriculum, and professional development needs, and 3) an audit of the schools’ PA facilities and play spaces. The site visit data were summarized into school-specific narratives that described the context, space/facilities, and the school assets and needs. Accuracy of the narratives was verified by school personnel [30]. Inductive content analysis was used to explore common strengths and areas of need across sites [31-33]. All aspects of this investigation were approved by West Virginia University (WVU) Institutional Review Board (IRB) (CHOICES protocol # 1301010629; CARDIAC protocol # 14244). All adult data collection in this assessment was based on voluntary participation. Due to the benign nature of the questions in the student surveys a waiver of signed informed consent was permitted by the WVU IRB. Parents and caregivers were noted about the data collection with a 10 day notice and requested to contact the respective school to withdraw their child from the CHOICES data collection. CARDIAC data (BMI screenings) were conducted with informed signed parental/caregiver consent.

### Analyses and reporting

The Town Hall meetings questionnaire responses were analyzed quantitatively and supported by field notes from the group discussion related to questionnaire response. The online PA interest survey responses were analyzed using Survey Monkey analytical tools [34]. The quantitative analysis of the Student PA and Health-related Behavior Survey was conducted using descriptive statistics, frequency tables and cross tabulations with SPSS 21. Student BMI data was analyzed using the CDC BMI growth charts, and reported in the four categories of underweight, normal weight, overweight, and obese. Results of the focus groups were analyzed using a qualitative analysis using the SWOT framework. Findings from the school site visits were collectively compiled by school and reported to the school administrators for verification in a narrative format.

All interview and focus group protocols as well as survey questionnaires have been made available to interested readers and uploaded as Additional files 1, 2, 3, 4, 5 and 6 with this paper.

## Results

### Town hall meetings

The main findings from the two THM were that all respondents (100%) somewhat or strongly agreed that it would be beneficial to have PA opportunities available at local public school sites in the evenings and weekends, and 97% stated such activities would be well received at senior centers. On the other hand, 82% of respondents in THM had not used an indoor school facility to be physically active in the last year and 75% had not used an outdoor school facility to be physically active within the last year. Almost all agreed that if a suitable recreational or PA activity facility was located close to their home they would use it at least once per week. All respondents agreed that more children’s after school PA programs are needed in McDowell County as well as more summer PA programs and clubs. About 95% stated they would participate in an organized evening PA class if made available to them. An interesting outcome was that 92% of the attendees had access to the Internet regularly either in their homes or somewhere else. An important take home message from these meetings was that although most people live in close proximity to a school, the school facilities were not used, or were not accessible, to the public for PA participation, but citizens believe that it would be beneficial and would be used by the community members. It was also agreed that new and improved outdoor play spaces are needed in McDowell County, and that children need more opportunities for structured and unstructured play, including after school and summer programs, and community-based programs.

### Physical activity interest survey

In order to help determine what types of physical activities are most appealing to the children and youth of McDowell County, we asked teachers to administer an online survey during health classes. Table 1 shows the 10 most commonly mentioned types of activities that students in McDowell County would be interested in being offered to them. Additionally, the PA Interest Survey included questions about feelings towards PA more generally. Over 90% stated interest in some of the activities listed and 78% would participate in an after school PA program with one of those activities if made available to them.

**Table 1:** **The 10 highest scoring physical activities (out of 35 possible items)**

**Activity**	**Mean (span =1-4)**	**% Highest score of importance (4)**
Swimming	3.44	64.8
Archery	3.33	58.1
Bowling	3.26	55.3
Basketball	3.10	51.9
Active gaming	3.15	50.4
Climbing wall	3.13	44.1
Kayaking	3.02	39.8
Softball	2.78	39.8
Mountain biking	2.94	38.0

### Student PA and health-related behavior survey

Results associated with current levels of PA and/or inactivity among 5^th^ and 8^th^ grade children in McDowell County are shown it Table 2. Around a third of boys and even fewer girls in the county meet the national guidelines of PA of 60 minutes or more per day. About one-third of the children in 5^th^ grade attend physical education every day. Sedentary lifestyles are common among these children as indicated in their technology usage. In addition, the students’ behavioral intentions for the future are reflective of their current levels of PA as shown in the table.

**Table 2:** **Student PA and health-related behavior survey**

	**5** ^**th**^ **Grade (%)**		**8** ^**th**^ **Grade (%)**	
	**Boys**	**Girls**	**Boys**	**Girls**
Physically active for at least 60 minutes every day	34.6	29.6	34.9	14.3
Attends PE classes at least 5 times in an average week	36.1	35.7	69.9	61.6
Definitely will be physically active for at least 60 min tomorrow	33.6	38.8	51.9	24.7
Definitely will be physically active for at least 60 minutes per day during 5 days or more next week	25.0	34.0	33.3	17.8
Plays video/computer games for 3 or more hours on an average school day	43.5	42.8	39.8	41.7
Watches TV for 3 hours or more on an average school day	36.1	40.8	32.5	36.3

Selected results (N = 442).

### CARDIAC BMI screening

Of the 550 students screened in the second and fifth grades, 52.9% overall were above the 85^th^ percentile (overweight/obese) and 57.0% of 5^th^ graders were overweight/obese, compared to the WV average of 46.9% while 48.1% of 2^nd^ graders were overweight/obese, compared to 38.5% statewide.

### Focus groups

Generally the results from the two sets of focus groups outlined in Table 3 (school- and community personnel) mirror each other. Children, youth and adults in McDowell County are generally interested in PA. Schools were the most commonly mentioned indoor facilities that might be made available to the public in this respect and McDowell County also has several parks and other outside spaces for PA. Some participants highlighted concerns about the access to the schools for students and other citizens outside school hours; “..you’ve got all these nice schools with nice gymnasiums and try to get them. It’s like having an audience with the Pope to try to get to use the school facility paid for by taxpayers’ money” said one participant. Liability issues were mentioned as one possible reason for difficulty in acquiring access to school facilities for PA outside school hours. Several participants signaled a need for renovation and improved lighting in outside spaces and parks. A need for park attendants was also mentioned.

**Table 3:** **Summary of focus group results for the McDowell CHOICES program**

**SWOT**	**School focus groups**	**Community focus groups**
**Strengths**	1. General interest in PA is high and participation in the few available programs is usually good.	1. General interest in PA is high among children, adolescents and adults.
	2. School infrastructure/facilities.	2. School facilities.
		3. Several outside spaces and county and state parks.
**Weaknesses**	1. Widespread sedentary lifestyle of children.	1. Poor condition of existing outside spaces and parks.
	2. Lack of access to proximal PA resources.	2. Geographical size of McDowell County relative to number of inhabitants.
	3. Multigenerational families (grandparents and great grandparents raising children).	3. Long travel time.
		4. Poor promotion of existing PA opportunities.
	4. Lack of appropriate, safe and accessible facilities for PA.	5. Lack of suitable platforms for communication.
	5. General narrow view and sheltered perspective of PA possibilities.	6. Absence of suitable indoor facilities for the winter months.
**Opportunities**	1. Citizens generally open to PA opportunities, especially around the schools.	1. People generally open towards new PA opportunities.
	2. Conversion/renovation of existing school facilities.	2. A web-based platform with coordinated information of PA opportunities.
	3. Shared use agreements for the use of school PA facilities for students and families.	3. Several smaller scale PA rather than centralized larger ones.
	4. Community fitness centers.	4. Renovation of existing facilities a sensible starting point.
	5. Need to train local people.	
**Threats**	1. Poverty.	1. Poverty.
	2. Locality/distrust of outsiders.	2. Multigenerational/aging families.
	3. Multigenerational/aging families.	3. Prevalent drug use/abuse, especially with prescription drugs.
	4. Poor condition and rampant vandalism of existing infrastructure/facilities.	4. High unemployment rate.
	5. Prevalent drug use/abuse	5. Geographic isolation.
	6. Poor road condition.	6. Poor travel/road conditions.
	7. Social capital/suitable professionals to step up and run new PA programs.	

All focus group participants agreed that sedentary lifestyle is a widespread problem in the county. A commonly mentioned reason for this was problems with access to proximal PA recourses relative to number of inhabitants in the county (population density: 41/sq mi). In this respect, the geographic spread and long distances/long travel time in McDowell County represents a macro level problem that adds on the issue of concentration of general poverty in the area.

Most participants highlighted a need for increasing the availability of localized PA opportunities. On the other hand lack of promotion of existing programs was also highlighted as a concern. A web-based platform with both existing and planned future programs was suggested as a possible solution that would work for most people given that over 90% of the citizens have access to internet in their homes or in close proximity to their homes.

### School site visits

Based on the cross-case analysis of the site narratives, six themes and key findings related to county-wide school PA environments and opportunities emerged (Table 4). The themes provided insight to both the strengths and limitations across all sites relative to factors impacting the design and implementation of CSPAPs. The six themes included: a) leadership and capacity building; b) PA access and opportunities; c) PE/PA equipment and resources; d) physical fitness data management and reporting; e) equity and access to safe and usable play spaces; and f) community connections. Table 4 summarized the themes’ highlights for improving PA promotion at the school level.

**Table 4:** **Summary of school site visit themes**

**Theme**	**Summary of theme**
Leadership and capacity building	Individuals or groups within the school are needed to champion PA opportunities. School level PA leaders can build and develop connections with families, school personnel, and the community.
PA access and opportunities	Efforts to integrate physical activity throughout the school day – before, during, and after – are important.
PE/PA equipment and resources	Additional equipment, resources, and materials for teachers to deliver a quality PE curriculum and a variety of other physical activity programming is needed.
Physical fitness data Management and Reporting	The use of a standardized method of fitness testing, data collection and reporting is needed.
Equity and access to safe and usable play space	Indoor physical space where children can be active during the school day is adequate and often times optimal, however outdoor play spaces often lack maintenance and upkeep.
Community connections	Existing or potential partnerships with community members or organizations with physical activity goals should be optimized, including shared use agreements with the schools.

## Discussion

The findings of this comprehensive needs assessment study indicate that both children and adults of McDowell County want to be physically active and would use facilities and participate in PA programming if made available to them. Further, the SWOT analysis of focus group material suggest that the citizens of McDowell County are generally open towards new PA ideas and that a central communication (e.g. internet-based) platform would be beneficial to advertise new programs and boost participation. This is particularly important in the light of community engaged research which has shown that communities differ in their readiness to engage in partnerships to promote community health [35,36]. On the other hand the focus group findings also point towards several structural barriers to increasing PA such as the geographic size of McDowell County relevant to population size, as well as high rates of poverty and a large proportion of families where grandparents are raising children. Additionally, the survey findings show that currently only about a third of boys and even fewer girls meet the national PA guidelines of 60 minutes or more per day. Sedentary lifestyles are also very common among children in McDowell County with close to 40% of children in 5^th^ grade and around 35% of 8^th^ graders watching TV every day for at least 3 hours and about 43% of 5^th^ grade children and 40% of 8^th^ graders playing video/computer games for at least 3 hours every day. Similar findings have been reported in the same age group in other rural parts of the US [37]. The CARDIAC screening also shows the prevalence of overweight and obesity is higher in McDowell County than the rest of WV. Results from the school site visits suggest that schools may serve as a potential platform for change. We now relate our findings to the three key concepts put forth in the introduction; issue selection, community capacity, and empowerment.

### Issue selection

Although the two THM were not carried out with the same research rigor typically needed in predictive models, they overwhelmingly suggest that PA opportunities are an important issue for the citizens of McDowell County. In addition to these meetings of the citizens, although not a formal part of this study, a meeting with key stakeholders (e.g., County Delegate, Superintendent of Schools) in the county prior to the start of the needs assessment planning revealed that the county leaders also enthusiastically support PA promotion, and are willing to work to increase PA opportunities. The results of the student PA interest survey point in the same direction, with over 90% of respondents stating interest in some activities surveyed. The Student Health-related Behavior Questionnaire also states a clear need for more PA opportunities given the high rates of sedentary activities and low rates of individuals being physically active for at least 60 min per day. The rates of overweight and obesity as revealed in the CARDIAC BMI screening results reiterate this need. The SWOT analyses of the focus group material also shows that general interest in PA is high and that citizens welcome new opportunities for PA but that poor conditions of available structures (e.g. school facilities, outside spaces/parks) are barriers to increased PA participation. Finally, the school site visits show efforts to integrate PA across the school day and beyond are needed to raise awareness and increase PA participation.

### Community capacity

THM attendees responded positively to a need for more summer PA programs and clubs. Given the rural and locality driven nature of such programs and clubs, local individuals would be ideally positioned to run such programs. Findings from the two surveys also underline the need to increase the community capacity to run programs and sustain both formal and informal PA opportunities. The results from the focus groups point toward the schools as formal infrastructure that might be used in this capacity. Because of the geographic size of McDowell County (population size/long travel distances) it was repeatedly pointed out that several smaller programs might work better to increase participation in PA rather than a single or two centralized opportunities. In this respect, all schools in McDowell County contain indoor gymnasiums that through shared use agreements might be opened up to the general community to engage in informal PA. This would also take care of the need for suitable indoor facilities over the winter months. Representatives from other school and community programs with an interest in improving health and reducing health risks agreed that working together for the common cause would be most beneficial and sustainable. Mayors of many small McDowell communities proposed solutions that would improve PA opportunities in their contextual environment. School site visits revealed that school leaders are willing to focus more on PA and work with their school personnel to develop a comprehensive school physical activity plan that would serve as a guide for providing more PA opportunities throughout the school day and beyond.

### Empowerment

Empowering citizens of McDowell County to take ownership of improved PA opportunities seems straightforward given the findings from all the needs assessment measures with school and community leaders and citizens as well as with students across the grades [25]. Training local residents to be PA leaders in after school programs and other community programs is an important step in McDowell County taking control of new and available PA opportunities. This echoes the meta-volition model [38] where organizational leadership is identified as the most critical element in the transition of communities towards a more physically active lifestyle. Establishing programs with dedicated leaders that live and work in McDowell County is empowering to the people, and can facilitate sustainability and capacity building within the county groups and organizations to work towards change. The passage of the McDowell County School Public Schools’ county-wide community collaborative innovation zone plan by the WV Board of Education in June 2013 allows the schools to be used as Community Schools and can help provide structure to support enhanced school-based PA opportunities [31]. The findings of this needs assessment also suggest that establishing shared use agreements to use school facilities may be particularly helpful as well, and support for acquiring new PA equipment and materials to use in the school setting will strengthen PA promotion [31]. As more emphasis is placed on PA and health-enhancing practices, local residents will become more aware of new opportunities for PA and this new knowledge and experiences are likely to serve to empower them to embrace such opportunities [22]. Community leaders and local citizens also expressed a desire to change the environment through renovation of existing indoor and outside spaces that might only need cleaning up to become functional for PA. In addition, setting up a centralized platform of communication, preferably online, would serve as important avenue of shared interest.

### Strengths and limitations of the needs assessment

The community needs assessment reported on in this paper has several limitations as well as some strengths. First of all, all aspects of this data collection and reporting are limited to McDowell County in WV and therefore not generalizable to other parts of the US. Our approach was to utilize the toolbox of methods available to the social and behavioral health sciences that can be specifically applied to each unique study. This context-specific and applied approach has been outlined by Livingood et al. [39]. Second, it is possible that the PA interest survey may possess biased estimates as all children that were interested in participating were allowed to respond to the survey. The results should be interpreted with caution in this respect as they are based on a convenience sample of interested participants. Third, our analysis of the semi-structured focus group data was unusual in the sense that we were directive to the SWOT sub-groups and decided to largely ignore other information collected in this part of the data collection. It of course would be possible to code and analyze the focus group data differently and come up with somewhat different results. Lastly, because our youngest study participants are 5^th^ graders we did not define levels of PA in the surveys as “moderate to vigorous” which is the standard definition in the PA literature [40]. Responses to the question on 60 minute daily PA may therefore not be as accurate as if reported for moderate to vigorous activity. A particular strength of this needs assessment is the comprehensiveness of the data assembly that engaged a large number of participants from many sectors in the community in McDowell County using several forms of data collection.

## Conclusion

West Virginia has a statewide physical activity plan that was developed by stakeholders representing all societal sectors and all geographic regions within the state. The Plan is dedicated to the promotion of physical activity opportunities and participation and the five priorities include 1) building capacity between schools and communities, 2) facilitating change through public awareness and marketing, 3)engaging communities in environment modifications to help facilitate increased physical activity, 4) sharing the responsibility of support for physical activity among a wide range of organizations, and 5) networking to make policy changes that has potential to influence physical activity accessibility [41-43]. The results of this study indicate that McDowell County residences, communities, and schools are dedicated to the priority areas of the West Virginia physical activity plan (WVPAP), and are ready to implement strategies to improve physical activity opportunities and participation. Results revealed both strengths and limitations that relate to the priority areas, but show a desire to make changes to improve the health of McDowell County citizens. The citizens of McDowell County are generally interested in PA and motivated to increase their levels of PA and opportunities. This study shows evidence of multi-sector collaboration that is possible within the county, and its findings will serve to assist in the development of programs that target this specific contextual environment and can potentially affect positive behavior changes.

## Additional files

Additional file 1:
**Script for SWOT focus group needs assessment with Community Stakeholders.**
Additional file 2:
**Script for SWOT focus group needs assessment with School Personnel.**
Additional file 3:
**McDowell CHOICES Town Hall Meeting Questions.**
Additional file 4:
**McDowell County CHOICES Student Physical Activity Interest Survey.**
Additional file 5:
**McDowell CHOICES QUESTIONNAIRE.**
Additional file 6:
**McDowell CHOICES Planning Project.**

## Abbreviations

BMI: Body mass index
CARDIAC: Coronary artery risk detection in Appalachian communities
CDC: Centers for disease control and prevention
CHOICES: Coordinated health opportunities involving communities environments, and schools
CSPAP: Comprehensive school physical activity programs
FERPA: Family Educational Rights and Privacy Act
PA: Physical activity
PE: Physical education
SWOT: Strengths weaknesses, opportunities, threats
THM: Town Hall meetings
WV: West Virginia
