# Supplementary material for: Needs assessment of school and community physical activity opportunities in rural West Virginia: the McDowell CHOICES planning effort
Source: BMC Public Health. 2015 Apr 3;15:327. doi: 10.1186/s12889-015-1702-9 (PMC4423593; doi:10.1186/s12889-015-1702-9)
Supplement: Additional file 1: — Script for SWOT focus group needs assessment with Community Stakeholders. [file 12889_2015_1702_MOESM1_ESM.docx]

**McDowell CHOICES.**

**Script for SWOT focus group needs assessment with Community Stakeholders.**

**Introduction for participants:**

**Moderator reads:**

Thank you for taking time to participate in the focus group with us. My name is ______ and I am __________ at West Virginia University. I am here as a part of the McDowell CHOICES project team to gain understanding from you about how best we may be able to serve McDowell County and its citizens in our project.

The McDowell CHOICES is a 2 year potentially renewable community project funded by the Highmark Foundation with the aim of improving the opportunities and conditions for participation in formal and informal physical activity.

The role of this focus group is therefore for us, the project conductors, to gain insight and understanding from you, the citizens of McDowell county, into the current strengths and weaknesses, and potential future opportunities and threats, around physical activity in the area.

I will ask you a number of open questions about the strengths, weaknesses, opportunities and threats to physical activity and how it relates to your work in the community. There are no right or wrong answers. I ask you to focus on strengths when I ask about strengths, on weaknesses when asked about those, and so on.

The focus group will be recorded [show recorder] and I ask you to talk as clearly as you can, so the recorder will be able to pick up everything that is said. Please try not to interrupt each other so we do not miss out on anything. Although I sit here with you and hear your thoughts and answers, your responses will be kept completely anonymous and nothing will be repeated after you as individuals. Your answers will be summarized with answers from the other participants and focus groups. Afterwards, the records will be transcribed to a readable computer file such as Microsoft Word, and thereafter the recordings will be erased.

You have important knowledge and understanding of the community that we, who are outsiders, do not have. We therefore greatly benefit from your input and we thank you dearly for taking part in this group today.

**Moderator writes**: Please indicate the number of participants: Male______ Female_______

-------

**Question sessions - STRENGTHS**

**Moderator reads:** If I can ask you first to focus on the current STRENGTHS in McDowell county.

1. If we think about physical activity, individual people and strengths. What are the current STRENGTHS in the community with regards to individuals in the area?

**Probe:**

-Do individuals generally have a preferred type of physical activity or sport?

-Are there particular individuals that are enthusiastic about physical activity or certain type of sports? (what kind of sports).

1. What about the families in the area. What are the current STRENGTHS in the community with regards to the families and physical activity in the area?

**Probe:**

-Are there particular family type physical activities that are common in the area?

-Are there certain families around that are enthusiastic about physical activity or certain type of sports?

1. If we now think about existing physical activity programs and STRENGTHS. Are there any current popular physical activity programs in the community?

**Probe:**

-What do you see as strengths of the existing programs?

-Is there an enthusiastic leader/instructor in the program(s)?

1. And now, if we think about indoor facilities and physical activity. What are the current STRENGTHS for physical activity with regards to indoor facilities?

**Probe:**

-Are indoor facilities for physical activity generally available?

-Are indoor facilities for physical activity generally useable?

1. If we think about outdoor facilities and physical activity. What are the current STRENGTHS for physical activity with regards to outdoor facilities?

**Probe:**

-Are outdoor facilities for physical activity generally available?

-Are outdoor facilities for physical activity generally useable?

1. And lastly, if we think about transport and distances and physical activity. What are the current STRENGTHS for physical activity with regards to transport or geographic distances?

**Probe:**

-Do people generally live close to areas where it is possible to be physically active?

-People live close to one another?

**Question sessions - WEAKNESSES**

**Moderator reads:** If I can ask you now to focus on the current WEAKNESSES in McDowell county.

1. If we think about physical activity, individual people and weaknesses. What are the current WEAKNESSES in the community with regards to individuals in the area?

**Probe:**

-Are individuals generally not interested in physical activity or sports?

-Are there very few leading individuals that are enthusiastic about physical activity or certain type of sports?

1. What about the families in the area. What are the current WEAKNESSES in the community with regards to the families and physical activity in the area?

**Probe:**

-Are there few or no family type physical activities that are common in the area?

-Are there no families around that are enthusiastic about physical activity or certain type of sports?

1. If we now think about existing physical activity programs and WEAKNESSES. What are the weaknesses of existing physical activity programs?

**Probe:**

-Is there not an enthusiastic leader/instructor available?

1. And now, if we think about indoor facilities and physical activity. What are the current WEAKNESSES for physical activity with regards to indoor facilities?

**Probe:**

-what are the weaknesses of available indoor facilities for physical activity?

-is access a problem?

1. And now, if we think about outdoor facilities and physical activity. What are the current WEAKNESSES for physical activity with regards to outdoor facilities?

**Probe:**

-what are the weaknesses of available outdoor facilities for physical activity?

-is access a problem?

1. And lastly, if we think about transport and distances and physical activity. What are the current WEAKNESSES for physical activity with regards to transport or geographic distances?

**Probe:**

-Do people generally live too far from areas where it is possible to be physically active?

-People live too far from one another to be active together?

**Question sessions - OPPORTUNITIES**

**Moderator reads:** If I can ask you now to think about future OPPORTUNITIES in McDowell county.

1. If we consider physical activity, the citizens and OPPORTUNITIES. Where do you see the future OPPORTUNITIES in the community with regards to individual people in the area?

**Probe:**

-Can people be encouraged to partake in physical activity or sport? How?

-Are there particular individuals that can be motivated towards some specific type of physical activity or sport?

1. What about the families. What do you see as future OPPORTUNITIES with regards to the families in the area?

**Probe:**

-Are there some family type physical activities that you would see as more likely to be successfully increased than others? Which ones?

-Are there certain families around that could be motivated about physical activity or certain type of sports?

1. If we now think about future physical activity programs and OPPORTUNITIES. What do you see as future opportunities for physical activity programs in McDowell County?

**Probe:**

-Do you think people would want to attend offered programs?

1. And now, if we think about indoor facilities and physical activity. What are the current OPPORTUNITIES for physical activity with regards to indoor facilities?

**Probe:**

-are there available indoor facilities for physical activity?

-what kind?

-is access relatively easy?

1. If we now think about outdoor facilities and physical activity. What are the current OPPORTUNITIES for physical activity with regards to outdoor facilities?

**Probe:**

-are there available outdoor facilities for physical activity?

-what kind?

-is time and access not a problem?

1. And lastly, if we think about transport and distances and physical activity. What are the current OPPORTUNITIES for physical activity with regards to transport or geographic distances?

**Probe:**

-Is it easy to gather people together for participation in physical activity?**Question sessions - THREATS**

**Moderator reads:** If I can ask you now to think about future **THREATS** in McDowell county.

1. If we consider physical activity, the citizens and **THREATS**. Where do you see the future **THREATS** in the community with regards to individual people in the area?

**Probe:**

-Is it hard to encourage people to partake in physical activity or sport? Why?

1. What about the families. What do you see as future **THREATS** with regards to the families in the area?

**Probe:**

-Are there no or few family type physical activities that you would see as likely to be increased? Why?

-Are there no or few families around that could be motivated about physical activity or certain type of sports?

1. If we now think about future physical activity programs and **THREATS**. What do you see as future threats for physical activity programs in McDowell County?

**Probe:**

-Limited attendance? Why?

1. And now, if we think about indoor facilities and physical activity. What are the current **THREATS** for physical activity with regards to indoor facilities?

**Probe:**

-are the available indoor facilities not useable?

-is time and access a problem?

1. If we now think about outdoor facilities and physical activity. What are the current **THREATS** for physical activity with regards to outdoor facilities?

**Probe:**

-are the available outdoor facilities not useable?

-is time and access a problem?

1. And lastly, if we think about transport and distances and physical activity. What are the current **THREATS** for physical activity with regards to transport or geographic distances?

**Probe:**

-Is it hard to gather people together for participation in physical activity
