# Supplementary material for: Needs assessment of school and community physical activity opportunities in rural West Virginia: the McDowell CHOICES planning effort
Source: BMC Public Health. 2015 Apr 3;15:327. doi: 10.1186/s12889-015-1702-9 (PMC4423593; doi:10.1186/s12889-015-1702-9)
Supplement: Additional file 2: — Script for SWOT focus group needs assessment with School Personnel. [file 12889_2015_1702_MOESM2_ESM.docx]

**McDowell CHOICES.**

**Script for SWOT focus group needs assessment with School Personnel.**

**Introduction for participants:**

**Moderator reads:**

Thank you for taking time to participate in the focus group with us. My name is ______ and I am __________ at West Virginia University. I am here as a part of the McDowell CHOICES project team to gain understanding from you about how best we may be able to serve McDowell County and its citizens in our project.

The McDowell CHOICES is a 2 year potentially renewable community project funded by the Highmark Foundation with the aim of improving the opportunities and conditions for participation in formal and informal physical activity.

The role of this focus group is therefore for us, the project conductors, to gain insight and understanding from you, the citizens of McDowell county, into the current strengths and weaknesses, and potential future opportunities and threats, around physical activity in the area.

I will ask you a number of open questions about the strengths, weaknesses, opportunities and threats to physical activity and how it relates to your work in and around schools. There are no right or wrong answers. I ask you to focus on strengths when I ask about strengths, on weaknesses when asked about those, and so on.

The focus group will be recorded [show recorder] and I ask you to talk as clearly as you can, so the recorder will be able to pick up everything that is said. Please try not to interrupt each other so we do not miss out on anything. Although I sit here with you and hear your thoughts and answers, your responses will be kept completely anonymous and nothing will be repeated after you as individuals. Your answers will be summarized with answers from the other participants and focus groups. Afterwards, the records will be transcribed to a readable computer file such as Microsoft Word, and thereafter the recordings will be erased.

You have important knowledge and understanding of the community that we, who are outsiders, do not have. We therefore greatly benefit from your input and we thank you dearly for taking part in this group today.

**Moderator writes**: Please indicate the number of participants: Male______ Female_______

-------

**Question sessions - STRENGTHS**

**Moderator reads:** If I can ask you first to focus on the current STRENGTHS in McDowell county.

1. If we think about physical activity, the students and strengths. What are the current STRENGTHS in your schools with regards to students?

**Probe:**

Do students generally have a preferred type of physical activity or sport?

Are there particular students that are enthusiastic about physical activity or certain type of sports?

Are there some students that are always physically active and playful during recess?

1. What about the families of students. What are the current STRENGTHS in our schools with regards to the families of students in the area?

**Probe:**

Are there particular family type physical activities that are common in the area?

Are there families around your schools that are enthusiastic about physical activity or certain type of sports?

1. If we now think about the school environment, physical activity and strengths. What are the current STRENGTHS for physical activity with regards to the school environment?

**Probe:**

Are their certain time slots that are allocated to physical activity in your schools?

Is there an enthusiastic physical educaiton teacher/instructor in your school? (what about the one that teaches PE?)

What about school facilities for physical activity? Inside or outside?

1. And lastly, if we think about transport and distances and physical activity. What are the current STRENGTHS for physical activity with regards to transport or geographic distances?

**Probe:**

Do people generally live close to areas where it is possible to be physically active?

People live close to one another?

**Question sessions - WEAKNESSES**

**Moderator reads:** If I can ask you now to consider the current WEAKNESSES in McDowell county.

1. If we think about physical activity, the students and weaknesses. What are the current WEAKNESSES in your schools with regards to students in the area?

**Probe:**

Do students generally not have a preferred type of physical activity or sport?

Are there particular students that are less enthusiastic about physical activity or sports?

Are there some students that are never physically active or playful during recess?

1. What about the families of students. What are the current WEAKNESSES in our schools with regards to the families of students in the area?

**Probe:**

Are there no or few family type physical activities that are common in the area (e.g., walking, cycling, skating, etc.).

Are there no families around your schools that are enthusiastic about physical activity or certain type of sports?

1. Turning our attention to the school environment, physical activity and WEAKNESSES. What are the current WEAKNESSES for physical activity with regards to the school environment?

**Probe:**

Are there no or few designated time slots that are allocated to physical activity in your schools?

Is there no physical education teacher/instructor in your school? (what about the one that teaches PE?)

What about WEAKNESSES in current school facilities for physical activity? Inside or outside?

1. And lastly, if we think about transport and distances and physical activity. What are the current WEAKNESSES for physical activity with regards to transport or geographic distances?

**Probe:**

Do people generally live far from areas where it is possible to be physically active?

People live far from one another?

**Question sessions - OPPORTUNITIES**

**Moderator reads:** If I can ask you now to think about future OPPORTUNITIES in McDowell county.

1. If we consider physical activity, the students and OPPORTUNITIES. Where do you see the future OPPORTUNITIES in your schools with regards to students in the area?

**Probe:**

Can students be encouraged to partake in physical activity or sport? How?

Are there particular students that can be motivated towards some specific type of physical activity or sport?

1. What about the families of students. What do you see as future OPPORTUNITIES in our schools with regards to the families of students in the area?

**Probe:**

Are there some family type physical activities that you would see as more likely to be successfully increased than others? Which ones?

Are there certain families around your schools that could be motivated about physical activity or certain type of sports?

1. About the school environment, physical activity and OPPORTUNITIES. What are the current OPPORTUNITIES for physical activity with regards to the school environment?

**Probe:**

Are there any time slots that might be used for physical activity in your schools?

What about using current school facilities for physical activity? Inside or outside?

1. And lastly, if we think about transport and distances and physical activity. What are the current OPPORTUNITIES for physical activity with regards to transport or geographic distances?

**Probe:**

Could we for example bring people together that live close to one another to a place where it is possible to be physically active?

Can we make use of short distances?

**Question sessions - THREATS**

**Moderator reads:** If lastly I can ask you to think about future THREATS in McDowell county.

1. If we consider physical activity, the students and THREATS. Where do you see the future THREATS in your schools with regards to students in the area?

**Probe:**

Are there barriers to student encouragement to partake in physical activity or sport? What kind of barriers?

Are there particular students that can hardly be motivated towards certain type of physical activity or sport?

1. What about the families of students. What do you see as future THREATS in our schools with regards to the families of students in the area?

**Probe:**

Are there not many family type physical activities that you would see as successfully increased? Why so?

Are there certain families around your schools that could hardly be motivated about physical activity or certain type of sports?

1. About the school environment, physical activity and THREATS. What are the current THREATS for physical activity with regards to the school environment?

**Probe:**

Are there no or few time slots that might be used for physical activity in your schools?

What about the current school facilities for physical activity? Inside or outside? Are they not applicable to be used for physical activity?

1. Finally, if we think about transport and distances and physical activity. What are the current THREATS for physical activity with regards to transport or geographic distances?

**Probe:**

Do you foresee it to be hard to bring people together to a place where it is possible to be physically active?

Are roads or long distances a problem?
