# Supplementary material for: Needs assessment of school and community physical activity opportunities in rural West Virginia: the McDowell CHOICES planning effort
Source: BMC Public Health. 2015 Apr 3;15:327. doi: 10.1186/s12889-015-1702-9 (PMC4423593; doi:10.1186/s12889-015-1702-9)
Supplement: Additional file 3: — McDowell CHOICES Town Hall Meeting Questions. [file 12889_2015_1702_MOESM3_ESM.docx]

**McDowell CHOICES Town Hall Meeting Questions**

**Related to physical activity environment:**

- If you wanted to walk 2-3 miles for exercise, how far is it from your home to an appropriate safe place where you could to do?
  - 0-2 miles, 3-5 miles, 6-8 miles, more than 8 miles
- How far is it from our home to the nearest school?
  - 0-2 miles, 3-5 miles, 6-8 miles, more than 8 miles
- How many times have you used an indoor school facility to be physically active in the last year (excluding student)?
  - Never, once, 2-5 times, 6-10 times, 11 times or more
- How many times have you used an outdoor school facility to be physically active in the last year (excluding student)?
  - Never, once, 2-5 times, 6-10 times, 11 times or more
- The citizens of McDowell County would benefit from having more outdoor play spaces.
  - Strongly agree, agree somewhat, disagree somewhat, strongly disagree
- If a suitable outdoor recreational space was available within 2 miles of my home I would use it at least once a week to by physically active (i.e. to walk, run).
  - Strongly agree, agree somewhat, disagree somewhat, strongly disagree

**Related to physical activity opportunities:**

- I believe it would be beneficial to the citizens of McDowell County to have physical activity opportunities available at local public school sites in the evenings and weekends.
  - Strongly agree, agree somewhat, disagree somewhat, strongly disagree
- I believe it would be beneficial to the citizens of McDowell County to have physical activity opportunities available at senior centers.
  - Strongly agree, agree somewhat, disagree somewhat, strongly disagree
- I believe it would be beneficial to the citizens of McDowell County to have physical activity opportunities available in church buildings.
  - Strongly agree, agree somewhat, disagree somewhat, strongly disagree
- There is a need for more children’s afterschool physical activity programs and clubs in McDowell County.
  - Strongly agree, agree somewhat, disagree somewhat, strongly disagree
- I would participate in an organized evening physical activity class if it was available to me.
  - Strongly agree, agree somewhat, disagree somewhat, strongly disagree

**Other Questions:**

- Access to the Internet in your home?
- Access to the Internet on a regular basis somewhere else other than your home?
- How would you rate your level of PA?
