# Supplementary material for: Needs assessment of school and community physical activity opportunities in rural West Virginia: the McDowell CHOICES planning effort
Source: BMC Public Health. 2015 Apr 3;15:327. doi: 10.1186/s12889-015-1702-9 (PMC4423593; doi:10.1186/s12889-015-1702-9)
Supplement: Additional file 4: — McDowell County CHOICES Student Physical Activity Interest Survey. [file 12889_2015_1702_MOESM4_ESM.pdf]

McDowell County CHOICES Student Physical Activity Interest Survey

McDowell County Students:

Today you are being asked to help the McDowell CHOICES team to identify physical activities that you would most like to participate in during physical education (PE) class, other times during the school day, or after school (even somewhere in your community). Your responses will help us better provide physical activities that you will like to participate in and learn more about.

PART 1 *of the survey has 35 physical activities that you are asked to rate. For each of the activities, please assign your rating according to your feelings about participating in this activity in your physical education class, other times during the school day, or after school (even somewhere in your community). If you would like to learn more about the activity and participate in it, give it a high rating. If not, give it a low rating.*

Some of the physical activities may be new to you, but you will get an idea of the activity by the picture provided. Some of them you will be familiar with and may already participate in during or after school. If so, rate according to how you feel about the activity now.

Rating scale: No Way (1) Not that interested (2) Looks like fun (3) So Cool (4)

PART 2 *of the survey has 7 questions about your current physical activity and diet habits, and 4 questions to rate your feelings on a scale of 1-10.*

Please remember to read and follow the directions carefully in each of the sections of the survey. You will not be able to complete the survey until you have answered every question – if you skip a question, the survey will return to that question before you can go on. At any time, you can choose to not complete the survey, in which case you simply exit from the survey by closing out the site. None of your answers will be saved or collected.

You may choose not to participate in this online physical activity interest survey. Participation or non-participation in the survey will not affect your grades in any way. We will not be collecting any names or personal information during this survey, so you can be sure that your responses are anonymous.

This survey is being conducted through West Virginia University College of Physical Activity and Sport Sciences as part of a study to help identify the needs and desires of McDowell County citizens that will help promote physical activity. This research has been approved and verified by the WVU Institutional Review Board.

Once you are ready to begin, please do not talk to anyone around you while taking the survey, and if you have any questions, please raise your hand and your teacher will help you.

By clicking on the "Continue" button, you are agreeing to participate in the online survey that will help us to know what physical activities are of most interest to you.

McDowell County CHOICES Student Physical Activity Interest Survey

About Me

***1. Select your school:**

- ◯ Mount View High School
- ◯ Mount View Middle School
- ◯ River View High School
- ◯ Sandy River Middle School
- ◯ Southside K-8

***2. Select your grade:**

- ◯ 6th Grade
- ◯ 7th Grade
- ◯ 8th Grade
- ◯ 9th Grade
- ◯ 10th Grade
- ◯ 11th Grade
- ◯ 12th Grade

McDowell County CHOICES Student Physical Activity Interest Survey

Rate the activity - Set 1

For each of the following groups of activities, assign your rating according to your feelings about participating in these activities in your PE class, other times during the school day, or after school (even somewhere in your community). If you would like to learn more about the activity and participate in it, give it a high rating. If not, give it a low rating.

There are seven groups of activities to rate (35 in all). Some of them may be new to you, but you will get an idea of the activity by the picture or you can ask your teacher to describe it for you. Some of them you will be familiar with and may already participate in. If so, rate according to how you feel about the activity now.

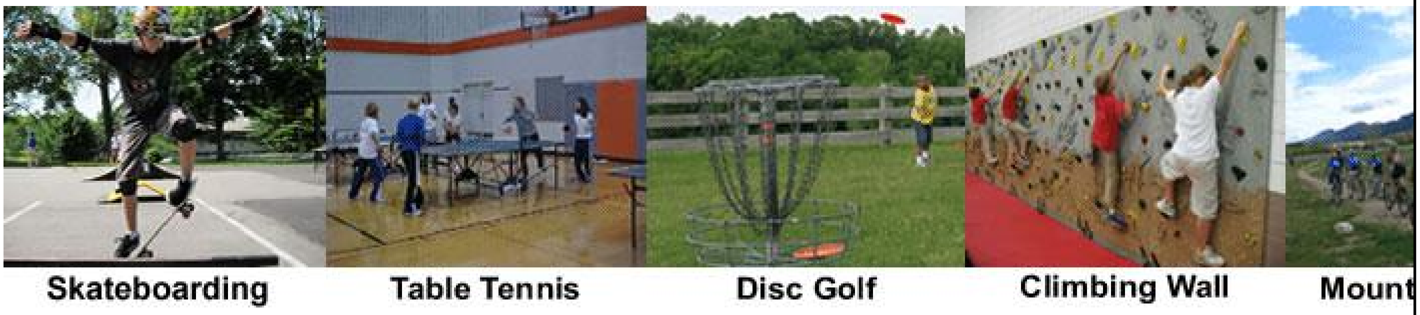

***1. Please rate the activities:**

	No Way	Not that interested	Looks like fun	So Cool
Skateboarding	◯	◯	◯	◯
Table Tennis	◯	◯	◯	◯
Disc Golf	◯	◯	◯	◯
Climbing Wall	◯	◯	◯	◯
Mountain Biking	◯	◯	◯	◯

McDowell County CHOICES Student Physical Activity Interest Survey

Rate the activity - Set 2

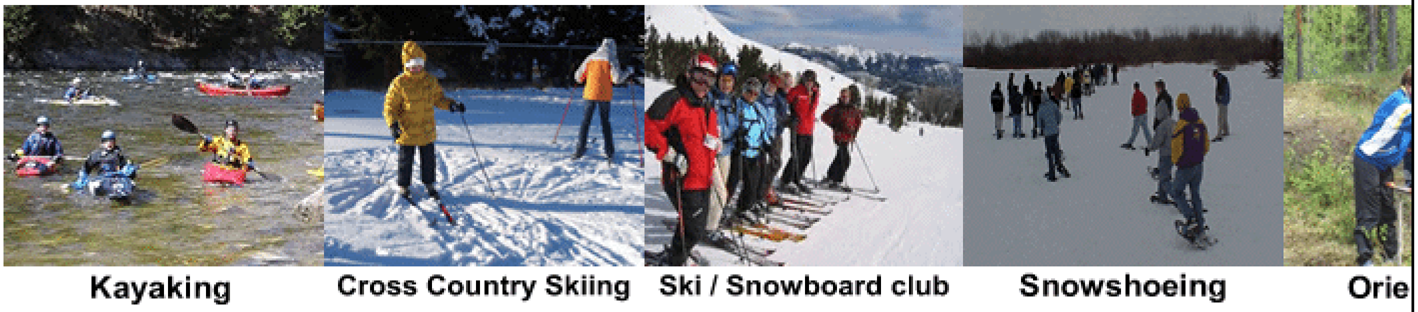

***1. Please rate the activities:**

	No Way	Not that interested	Looks like fun	So Cool
Kayaking	○	○	○	○
Cross Country Skiing	○	○	○	○
Ski/Snowboard Club	○	○	○	○
Snowshoeing	○	○	○	○
Orienteering	○	○	○	○

McDowell County CHOICES Student Physical Activity Interest Survey

Rate the activity - Set 3

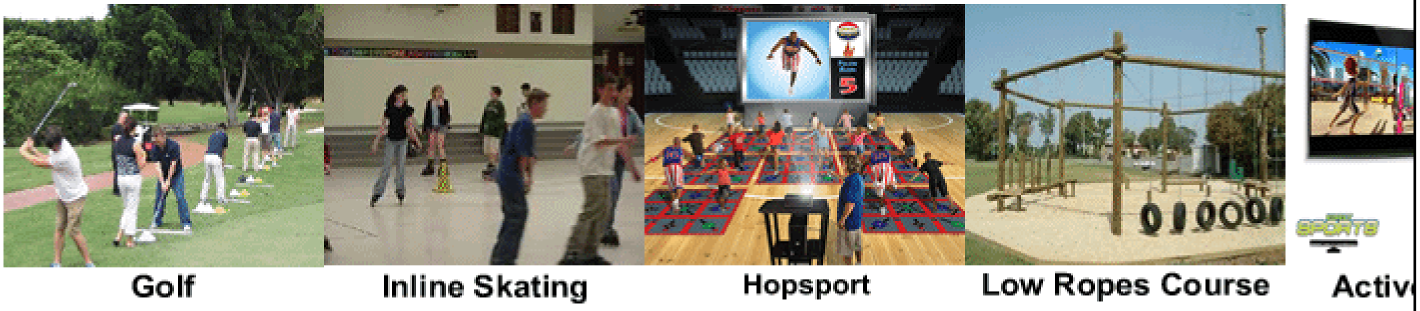

***1. Please rate the activities:**

	No Way	Not that interested	Looks like fun	So Cool
Golf	○	○	○	○
Inline Skating	○	○	○	○
Hopsport	○	○	○	○
Low Ropes Course	○	○	○	○
Active Gaming	○	○	○	○

McDowell County CHOICES Student Physical Activity Interest Survey

Rate the activity - Set 4

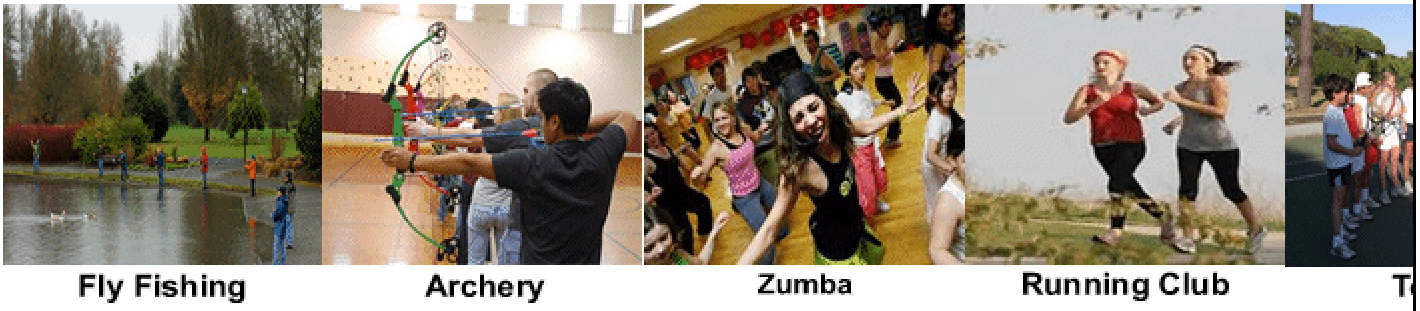

***1. Please rate the activities:**

	No Way	Not that interested	Looks like fun	So Cool
Fly Fishing	◯	◯	◯	◯
Archery	◯	◯	◯	◯
Zumba	◯	◯	◯	◯
Running Club	◯	◯	◯	◯
Tennis	◯	◯	◯	◯

McDowell County CHOICES Student Physical Activity Interest Survey

Rate the activity - Set 5

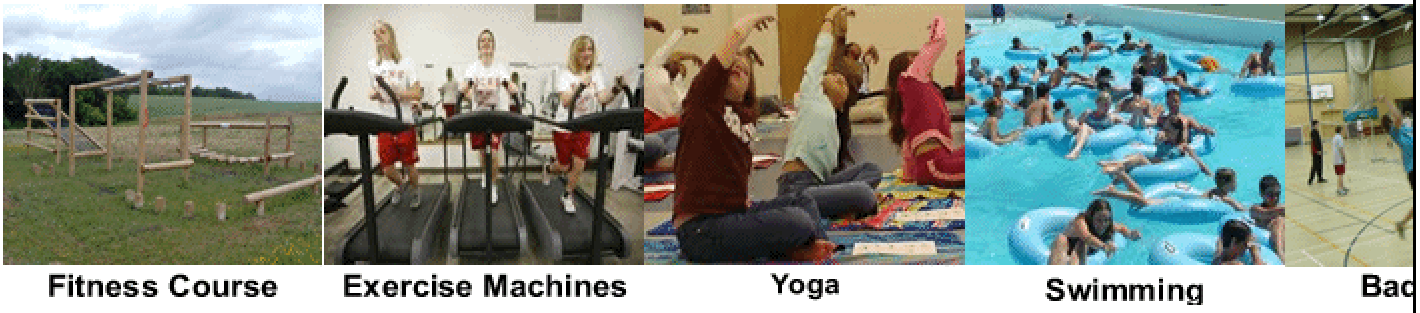

***1. Please rate the activities:**

	No Way	Not that interested	Looks like fun	So Cool
Fitness Course	○	○	○	○
Exercise Machines	○	○	○	○
Yoga	○	○	○	○
Swimming / Water Aerobics	○	○	○	○
Badminton	○	○	○	○

McDowell County CHOICES Student Physical Activity Interest Survey

Rate the activity - Set 6

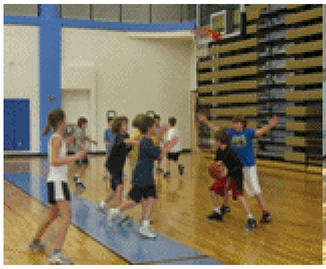
Basketball

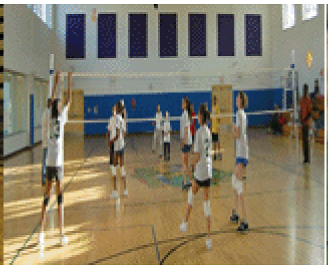
Volleyball

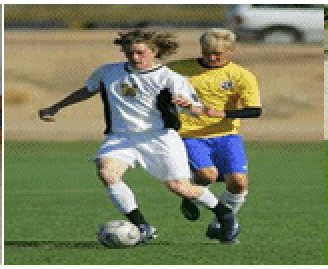
Soccer

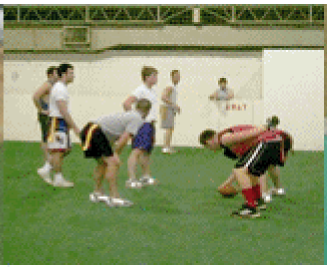
Flag Football

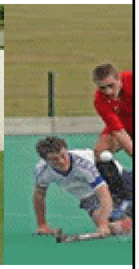
Fielc

***1. Please rate the activities:**

	No Way	Not that interested	Looks like fun	So Cool
Basketball	○	○	○	○
Volleyball	○	○	○	○
Soccer	○	○	○	○
Flag Football	○	○	○	○
Field Hockey	○	○	○	○

McDowell County CHOICES Student Physical Activity Interest Survey

Rate the activity - Set 7

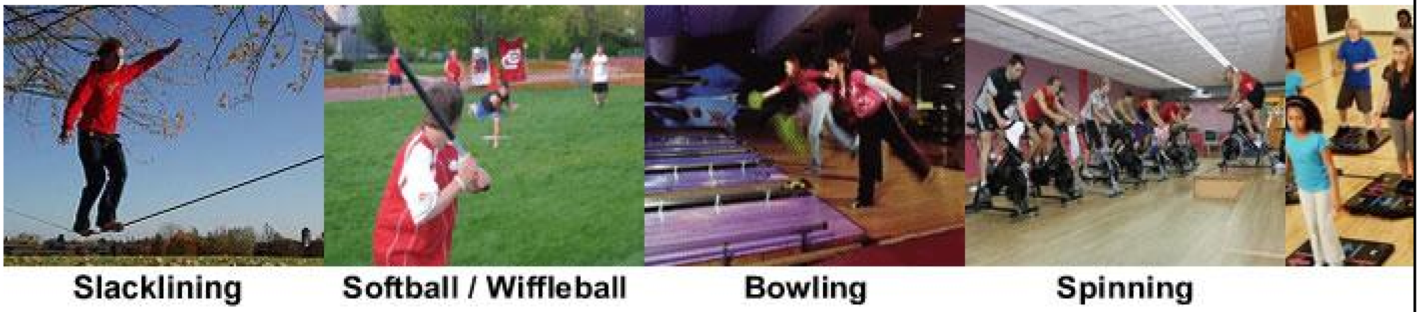

***1. Please rate the activities:**

	No Way	Not that interested	Looks like fun	So Cool
Slacklining	○	○	○	○
Softball / Wiffleball	○	○	○	○
Bowling	○	○	○	○
Spinning	○	○	○	○
Dance Dance Revolution (DDR)	○	○	○	○

McDowell County CHOICES Student Physical Activity Interest Survey

Rate your feelings

Please rate your feelings on each of the following questions

***1. If given the opportunity, I would like to participate in some of the physical activities listed above in my community in which I live.**

- Strongly Disagree
- Somewhat Disagree
- Somewhat Agree
- Strongly Agree

***2. If given the opportunity (transportation, etc) I would like to join an after-school club that involves some of these physical activities listed above.**

- Strongly Disagree
- Somewhat Disagree
- Somewhat Agree
- Strongly Agree

***3. I currently participate in physical activities at least 30 minutes a day on most days of the week.**

- Strongly Disagree
- Somewhat Disagree
- Somewhat Agree
- Strongly Agree

***4. I currently participate in physical activities at least 60 minutes a day on most days of the week.**

- Strongly Disagree
- Somewhat Disagree
- Somewhat Agree
- Strongly Agree

***5. I eat at least two fruits and three vegetables each day.**

- Strongly Disagree
- Somewhat Disagree
- Somewhat Agree
- Strongly Agree

***6. In addition to PE, I also participate in other physical activities during the school day (do NOT include school sports teams)**

- Strongly Disagree
- Somewhat Disagree
- Somewhat Agree
- Strongly Agree

***7. The national recommendation for the MINIMUM amount of physical activity that I should participate in daily is**

- 30 Minutes
- 45 Minutes
- 60 Minutes
- 90 Minutes

***8. Please rate your feelings on each of the following:**

	Lowest (1)	2	3	4	5	6	7	8	9	Highest (10)
Physical Education	○	○	○	○	○	○	○	○	○	○
Physical Activity, in general	○	○	○	○	○	○	○	○	○	○
Eating Fruits	○	○	○	○	○	○	○	○	○	○
Eating Vegetables	○	○	○	○	○	○	○	○	○	○

McDowell County CHOICES Student Physical Activity Interest Survey

Thank you for taking the Physical Activity Interest survey for the McDowell CHOICES Project! If you have any questions or concerns, do not hesitate to contact the McDowell CHOICES Team leader at 304-293-0869
