# Supplementary material for: Needs assessment of school and community physical activity opportunities in rural West Virginia: the McDowell CHOICES planning effort
Source: BMC Public Health. 2015 Apr 3;15:327. doi: 10.1186/s12889-015-1702-9 (PMC4423593; doi:10.1186/s12889-015-1702-9)
Supplement: Additional file 5: — McDowell CHOICES QUESTIONNAIRE. [file 12889_2015_1702_MOESM5_ESM.docx]

**McDowell CHOICES**

**QUESTIONNAIRE**

**STUDENT NAME: ________________________________ (Please PRINT your full name on the line)**

**GRADE: 5^TH^□ 7^TH^□ 10^TH^□**

**SCHOOL:**

**□ #1**

**□ #2**

**□ #3**

**□ #4**

**□ #5**

**□ #6**

**□ #7**

**□ #8**

**□ #9**

**Student Physical Activity and Nutrition Survey**

**PLEASE COMPLETE ALL 8 PAGES IN THE BOOKLET BY MARKING ONE BOX FOR EACH QUESTION**

1. During the **past 7 days**, on how many days were you physically active for a total of **at least 60 minutes per day**? (Add up all the time you spent in any kind of physical activity that increased your heart rate and made you breathe hard some of the time.)

□ 0 days

□ 1-2 days

□ 3-4 days

□ 5-6 days

□ 7 days

1. On an **average school day**, how many hours do you watch TV?

□ I do not watch TV on an average school day

□ Less than 1 hour per day

□ 1-2 hours per day

□ 3-4 hours per day

□ 5 or more hours per day

1. On an **average school day**, how many hours do you play video or computer games or use a computer for something that is not school work? (Include activities such as Xbox, PlayStation, Nintendo DS, iPod touch, Facebook, and the Internet.)

□ I do not play video or computer games or use a computer for something that is not school work

□ Less than 1 hour per day

□ 1-2 hours per day

□ 3-4 hours per day

□ 5 or more hours per day

1. In an **average week** when you are in school, on how many days do you go to physical education (PE) classes?

□ 0 days

□ 1-2 days

□ 3-4 days

□ 5-6 days

□ 7 days

1. During the **past 12 months**, on how many sports teams did you play? (Count any teams run by your school or community groups.)

□ 0 teams

□ 1 team

□ 2 teams

□ 3 or more teams

1. **Yesterday**, how many times did you drink any kind of milk? (INCLUDE chocolate or other flavored milk, milk on cereal, and drinks made with milk.)

□ 0 times

□ 1 time

□ 2 times

□ 3 times

□ 4 or more times

1. **Yesterday**, how many times did you eat any orange vegetables like carrots, squash, or sweet potatoes?

□ 0 times

□ 1 time

□ 2 times

□ 3 times

□ 4 or more times

1. **Yesterday**, how many times did you eat a salad made with lettuce, or any green vegetables like spinach, green beans, broccoli, or other greens?

□ 0 times

□ 1 time

□ 2 times

□ 3 times

□ 4 or more times

1. **Yesterday**, how many times did you eat any other vegetables like peppers, tomatoes, zucchini, asparagus, cabbage, cauliflower, cucumbers, mushrooms, eggplant, celery or artichokes?

□ 0 times

□ 1 time

□ 2 times

□ 3 times

□ 4 or more times

1. **Yesterday**, how many times did you eat fruit? Fruits are all fresh, frozen, canned, or dried fruits. (DO NOT COUNT fruit juice.)

□ 0 times

□ 1 time

□ 2 times

□ 3 times

□ 4 or more times

1. **Yesterday**, how many times did you drink fruit juice? Fruit juice is a 100% juice drink like orange juice, apple juice, or grape juice. (DO NOT COUNT punch, Kool-Aid®, sports drinks, and other fruit-flavored drinks.)

□ 0 times

□ 1 time

□ 2 times

□ 3 times

□ 4 or more times

1. **Yesterday**, how many times did you drink any punch, Kool-Aid®, sports drinks, or other fruit-flavored drinks? (DO NOT COUNT 100% fruit juice or soda.)

□ 0 times

□ 1 time

□ 2 times

□ 3 times

□ 4 or more times

1. **Yesterday**, how many times did you drink any regular (NOT diet) sodas or soft drinks?

□ 0 times

□ 1 time

□ 2 times

□ 3 times

□ 4 or more times

1. **Yesterday**, how many times did you drink any diet sodas or soft drinks?

□ 0 times

□ 1 time

□ 2 times

□ 3 times

□ 4 or more times

1. **Yesterday**, how many times did you drink a bottle or glass of water? (INCLUDE sparkling or any other water drink that has 0 calories.)

□ 0 times

□ 1 time

□ 2 times

□ 3 times

□ 4 or more times

| **16..** | How likely is it that you will be physically active for 60 minutes tomorrow? |  |  |
| --- | --- | --- | --- |
|  | \|  \| □ I Definitely will not \| \| --- \| --- \| \|  \| □ I Probably will not \| \|  \| □ I Probably will \| \|  \| □ I Definitely will \| |  |  |
| \| **17.** \| How likely is it that you will be physically active 5 days next week, for at least 60 minutes a day? \| \| --- \| --- \| \|  \| \|  \| □ I Definitely will not \| \| --- \| --- \| \|  \| □ I Probably will not \| \|  \| □ I Probably will \| \|  \| □ I Definitely will \| \|  \| **18.** \| How likely is it that you will make healthy decisions tomorrow in order to help maintain or obtain a healthy weight? \| \| --- \| --- \| \|  \| \|  \| □ I Definitely will not \| \| --- \| --- \| \|  \| □ I Probably will not \| \|  \| □ I Probably will \| \|  \| □ I Definitely will \| \|  \| **19.** \| Do you **agree or disagree** with the following statement: It is important to be physically active. \| \| \| --- \| --- \| --- \| \|  \| \|  \| □ Strongly Agree \| \| --- \| --- \| \|  \| □ Somewhat Agree \| \|  \| □ Not Sure \| \|  \| □ Somewhat Disagree \| \|  \| □ Strongly Disagree \| \|  \| **20.** \| Do you **agree or disagree** with the following statement: My friends think that it is important that I am physically active every day. \| \| \| --- \| --- \| --- \| \|  \| \|  \| □ Strongly Agree \| \| --- \| --- \| \|  \| □ Somewhat Agree \| \|  \| □ Not Sure \| \|  \| □ Somewhat Disagree \| \|  \| □ Strongly Disagree \| \| \| \| **21.** \| Do you **agree or disagree** with the following statement: My parent(s) think that it is important that I am physically active each day. \| \| \|  \| \|  \| □ Strongly Agree \| \| --- \| --- \| \|  \| □ Somewhat Agree \| \|  \| □ Not Sure \| \|  \| □ Somewhat Disagree \| \|  \| □ Strongly Disagree \| \| \| \| **22.** \| Do you **agree or disagree** with the following statement: I could participate in more physical activity during the school day if I wanted to. \| \|  \| \|  \| □ Strongly Agree \| \| --- \| --- \| \|  \| □ Somewhat Agree \| \|  \| □ Not Sure \| \|  \| □ Somewhat Disagree \| \|  \| □ Strongly Disagree \| \|  \| \| \| **23.** \| Do you **agree or disagree** with the following statement: I could participate in more physical activity after school if I wanted to. \| \| \|  \| \|  \| □ Strongly Agree \| \| --- \| --- \| \|  \| □ Somewhat Agree \| \|  \| □ Not Sure \| \|  \| □ Somewhat Disagree \| \|  \| □ Strongly Disagree \| \| \|   **24**. Do you **agree or disagree** with the following statement: There are physical activity opportunities (outdoor and/or indoor) in my community that members of my family can choose to participate in (at my school facility or at other places in my community).   \| □ Strongly Agree \| \| --- \| \| □ Somewhat Agree \| \| □ Not Sure \| \| □ Somewhat Disagree \| \| □ Strongly Disagree \|   **25**. Do you **agree or disagree** with the following statement: I am happy with the number of parks/play areas for physical activity in my neighborhood/community.   \|  \| \|  \| □ Strongly Agree \| \| --- \| --- \| \|  \| □ Somewhat Agree \| \|  \| □ Not Sure \| \|  \| □ Somewhat Disagree \| \|  \| □ Strongly Disagree \| \| \| --- \| --- \| --- \| --- \| --- \| --- \| --- \| --- \| --- \| --- \| --- \| --- \|   **26**. Do you **agree or disagree** with the following statement: I would use the school’s sport fields and/or gyms to be physically active if they were available to me after school and during weekends.   \|  \| \|  \| □ Strongly Agree \| \| --- \| --- \| \|  \| □ Somewhat Agree \| \|  \| □ Not Sure \| \|  \| □ Somewhat Disagree \| \|  \| □ Strongly Disagree \| \| \| --- \| --- \| --- \| --- \| --- \| --- \| --- \| --- \| --- \| --- \| --- \| --- \|   **27**. Do you **agree or disagree** with the following statement: I would consider participating in a physical activity program AFTER SCHOOL if it was available to me.   \|  \| \|  \| □ Strongly Agree \| \| --- \| --- \| \|  \| □ Somewhat Agree \| \|  \| □ Not Sure \| \|  \| □ Somewhat Disagree \| \|  \| □ Strongly Disagree \| \| \| --- \| --- \| --- \| --- \| --- \| --- \| --- \| --- \| --- \| --- \| --- \| --- \|   **28**. The national recommendation for the **minimum** amount of physical activity that you should participate in each day is:   \|  \| \|  \| □ 30 minutes \| \| \| --- \| --- \| --- \| \|  \| □ 45 minutes \| \| \|  \| □ 60 minutes \| \| \|  \| □ 90 minutes \| \| \|  \|  \|   **29**. The national recommendation for the **minimum** servings of fruits and vegetables you should eat each day is:   \|  \| □ 0-1 Servings \| \| \| --- \| --- \| --- \| \|  \| □ 2-3 Servings \| \| \|  \| □ 3-4 Servings \| \| \|  \| □ 5-6 Servings \| \| \|  \|  \| \| \| --- \| --- \| --- \| --- \| --- \| --- \| --- \| --- \| --- \| --- \| --- \| --- \| --- \| --- \| --- \| --- \| --- \| --- \| --- \| --- \| --- \| --- \| --- \| --- \| --- \| --- \| --- \| --- \| --- \| --- \|   **30**. Are you a boy or a girl:   \|  \| □ Boy \| \| --- \| --- \| \|  \| □ Girl \| | | | |

**THANK YOU FOR YOUR PARTICIPATION**

**PLEASE RAISE YOUR HAND AND GIVE THE QUESTIONNAIRE TO YOUR TEACHER**
