# Supplementary material for: Needs assessment of school and community physical activity opportunities in rural West Virginia: the McDowell CHOICES planning effort
Source: BMC Public Health. 2015 Apr 3;15:327. doi: 10.1186/s12889-015-1702-9 (PMC4423593; doi:10.1186/s12889-015-1702-9)
Supplement: Additional file 6: — McDowell CHOICES Planning Project. [file 12889_2015_1702_MOESM6_ESM.pdf]

McDowell CHOICES Planning Project

School Site Visits Protocol

Purpose

The purpose of the school site visits is to formatively evaluate the school physical activity environments and opportunities in McDowell County, with aim of identifying school-specific Comprehensive School Physical Activity Program (CSPAP) strengths, weaknesses, and areas for improvement.

Proposed Outcomes

Identification of school-specific CSPAP strengths and areas for improvement related to physical activity environment, opportunities, and policies.

School-specific CSPAP action plans for the 2-year McDowell CHOICES project.

Site Visit Protocol

Conceptual Framework. The Social Ecological Model will serve as the conceptual framework for the McDowell CHOICES school site visits. Within this model, individual behaviors, attitudes, and values, are said to be influenced by the dynamic interactions and relationships among various personal, social, and environmental factors. Such factors extend across four diverse levels of influence – interpersonal (family, peers), organizational (school, church, health services), community (mass media, neighbors, industry), and societal (cultural norms, policies, laws). Specifically informed by this model, the site visit protocol will examine school-level organizational factors (i.e., environment, norms, organizational culture, incentives, etc.) that may influence student physical activity opportunities and participation.

Comprehensive School Physical Activity Programs (CSPAP) promote physical activity opportunities for students before, during, and after the school day. This approach aims to help students obtain at least the minimum requirements of daily physical activity (60 minutes) through strategic offering of physical activity opportunities across many facets of the school setting. A well-constructed and articulated CSPAP has the potential for enhancing the school culture and norms toward physical activity and healthy decision making. Comprehensive physical activity programming, as it is recognized as an ecological approach, will also inform the framework for the site visit protocol.

Site Visit Teams. Members of the McDowell CHOICES team and graduate students will serve as members of the site teams. A site visit team consists of two individuals who will spend one half day at each respective school conducting one-on-one and small group interviews with designated school-level stakeholders and observational checklists of facilities and play spaces. Site visit teams will be provided training related to interview protocol, observational checklists, and related site visit protocols/procedures.

During March 2013 three two-person site visit teams will travel to McDowell County to conduct ten (10) site visits across a one week timeframe. While at the school site visit teams will have scheduled 30-45 minute time blocks with representative individuals from the following selected groups: a) Principal and b) Physical educator. The principal and PE teacher will assist in identifying classroom teacher, parent, and student representatives for the site visit group interviews. Interview topics and select worksheets (e.g., equipment inventory and curriculum content) and interview topic areas will be sent electronically to respondents prior to the site visit. Site team members will be given a tour of the school building, grounds, and play/athletic spaces and facilities. A $200 thank-you gift (e.g., equipment for the PE classroom) will be given to the school at the conclusion of the site visit.

Once site visit data are analyzed and school-specific data are summarized, principals will receive a report of strengths and areas for improvement relative to physical activity environment, opportunities, and policies.

Site Visit Activities

One-on-One Interviews (30-45 minutes each)

School Principal –
(1) CSPAP Inventory with modified response options. The Comprehensive School Physical Activity Program Inventory (Appendix A) will be completed with the school principal in a structured interview format allowing for follow-up open ended questions. Site team members will keep field notes on the inventory based upon principal responses. Following the interview, site team members will debrief into an audio recording device to supplement field notes.

Physical Educators –
(1) CSPAP Inventory with modified response options. The Comprehensive School Physical Activity Program Inventory (Appendix A) will be completed with the school principal in a structured interview format allowing for follow-up open ended questions. Site team members will keep field notes on the inventory based upon principal responses. Following the interview, site team members will debrief into an audio recording device to supplement field notes;
(2) PE Equipment inventory. The PE Equipment Inventory Checklist (Appendix B) will be sent prior to site visit for PE teachers to complete. This information will help to determine existing and needed PE equipment and resources. The inventory will be collected by the site team members on day of visit;
(3) Curriculum content worksheet. The PE Curriculum Content Worksheet (Appendix C) will be sent prior to site visit for PE teachers to complete. This information will provide greater understanding the context and existent PE programming. The worksheet will be collected by the site team members on day of visit;
(4) Professional development recall and PD needs worksheet. The Professional Development Activities Worksheet (Appendix D) will be sent prior to the site visit for PE teachers to complete. This information will help clarify the need for professional development workshops for PE teachers. The worksheet will be collected by the site team members on day of visit.

Tour of School – Physical activity and athletic facilities

Facilities and Play Space Audit and Usage. The Facilities and Play Space Audit Worksheet (Appendix E) will be completed by site team members during a tour of school grounds, play spaces, and athletic facilities.

Comprehensive School Physical Activity Program Inventory

Your school…	Fully in Place	Partially in Place	Under Develop -ment	Not in Place	Don't Know	Notes
Has adopted a local school wellness policy that includes one or more goals for physical education.	**3**	**2**	**1**	**0**		
Has adopted a local school wellness policy that includes one or more goals for physical activity (e.g., recess, walk/bike to school, before/after school physical activity programs).	**3**	**2**	**1**	**0**		
Implements policy and practices that address the physical education goal(s) in your local school wellness policy.	**3**	**2**	**1**	**0**		
Implements policy and practices that address the physical activity goal(s) in your local school wellness policy.	**3**	**2**	**1**	**0**		
Has a wellness policy committee that includes at least one physical education/activity expert.	**3**	**2**	**1**	**0**		
**Section Totals**						

Physical Education	Fully in Place	Partially in Place	Under Develop -ment	Not in Place	Don't Know	Notes
Employs only state-certified physical education teachers to teach elementary, middle, and high school physical education.	3	2	1	0		
Employs an individual who is responsible for the district-wide physical education program (e.g., physical education coordinator).	3	2	1	0		
Provides elementary school students with at least 150 minutes per week of physical education in all grades.	3	2	1	0		If not all grades, please specify:
Provides middle school students with at least 225 minutes per week of physical education in all grades.	3	2	1	0		If not all grades, please specify:
Provides high school students with at least 225 minutes per week of physical education in all grades.	3	2	1	0		If not all grades, please specify:
Requires use of a physical education curriculum that is based on state and/or national physical education standards.	3	2	1	0		
Requires physical education teachers to assess students on motor/movement skills and knowledge of physical activity concepts.	3	2	1	0		

	Fully in Place	Partially in Place	Under Develop -ment	Not in Place	Don't Know	Notes
Requires health-related fitness testing for students (in all grades or certain grades) and the results to be collected and analyzed at the district level.	**3**	**2**	**1**	**0**		
Requires that students receive a passing grade in physical education that uses the same grading scale as other academic subjects.	**3**	**2**	**1**	**0**		
Requires that class sizes and student-teacher ratios in physical education be consistent with those of other academic subjects.	**3**	**2**	**1**	**0**		
Requires that physical education teachers are periodically evaluated for performance by an appropriate administrator, including review of planning, instructional practices, and student assessment.	**3**	**2**	**1**	**0**		
Provides annual professional development for physical education teachers that is directly related to their curricula.	**3**	**2**	**1**	**0**		
Provides adapted physical education to students with special needs as identified through an IEP, which is delivered by and/or with support from adapted physical education specialists.	**3**	**2**	**1**	**0**		

Provides funding for physical education curriculum, facilities, equipment and professional development.	**3**	**2**	**1**	**0**		
Actively seeks funding to support the physical education program through foundation grants, government grants, community partners, corporate partners, and similar.	**3**	**2**	**1**	**0**		
Only allows students to be temporarily excused from physical education class for medical reasons.	**3**	**2**	**1**	**0**		
**Section Totals**						
**Physical Activity During School**	**Fully in Place**	**Partially in Place**	**Under Develop-ment**	**Not in Place**	**Don't Know**	**Notes**
Requires that elementary school students are provided with at least 20 minutes of active recess during each school day in all grade levels.	**3**	**2**	**1**	**0**		
Requires that physical activity (including recess) not be withheld as punishment or for disciplinary reasons, nor used as punishment for inappropriate behavior.	**3**	**2**	**1**	**0**		
Requires that a maximum of 60 minutes (elementary school) or 90 minutes (middle and high school) of seat time is followed by 5-10 minutes of physical activity breaks.	**3**	**2**	**1**	**0**		Examples:

Requires that physical activity breaks are provided immediately prior to testing.	**3**	**2**	**1**	**0**		
Provides professional development to classroom teachers to support integrating physical activity into the classroom.	**3**	**2**	**1**	**0**		Dates and Type:
**Section Totals**						
**Physical Activity Before and After School**	**Fully in Place**	**Partially in Place**	**Under Develop -ment**	**Not in Place**	**Don't Know**	**Notes**
Employs an individual who is responsible for district-wide school-based physical activity (e.g., interscholastic sports, before/after school physical activity clubs and intramural sports, walk/bike to school).	**3**	**2**	**1**	**0**		
Offers a variety physical activity clubs and/or intramural sports that are available to all students.	**3**	**2**	**1**	**0**		Participation Rates:
Implements a Safe Routes to School program to encourage walking and biking to school.	**3**	**2**	**1**	**0**		
Offers a variety of interscholastic sports that are available to all students.	**3**	**2**	**1**	**0**		Participation Rates:
Requires all interscholastic sport coaches to be certified to coach.	**3**	**2**	**1**	**0**		
Requires training for all interscholastic sport coaches.	**3**	**2**	**1**	**0**		

Requires that a certified athletic trainer be present at all interscholastic sport practices and games.	**3**	**2**	**1**	**0**		
Implements an interscholastic sport participation policy that addresses academic eligibility and adherence to school and program rules.	**3**	**2**	**1**	**0**		
Provides appropriate and safe equipment and facilities for all sport functions, games, and practices.	**3**	**2**	**1**	**0**		
**Section Totals**						
**Staff Involvement**	**Fully in Place**	**Partially in Place**	**Under Develop -ment**	**Not in Place**	**Unsure**	**Notes**
Provides a district-wide school employee wellness program.	**3**	**2**	**1**	**0**		Participation Rates:
Employs an individual who is responsible for the district-wide school employee wellness program.	**3**	**2**	**1**	**0**		
**Section Totals**						

Family and Community Involvement	Fully in Place	Partially in Place	Under Develop-ment	Not in Place	Unsure	Notes
Requires all schools to make their outdoor physical activity facilities available for student, staff, and community member use outside of school hours when not being used for school clubs and sports teams.	**3**	**2**	**1**	**0**		
Engages in joint use agreements to share school and/or community physical activity facilities and/or encourages schools to enter into joint use agreements.	**3**	**2**	**1**	**0**		List:
Partners with community organizations that support and enhance the physical education/activity program.	**3**	**2**	**1**	**0**		List:
Allows parents/guardians to observe physical education classes after proper sign-in at the school.	**3**	**2**	**1**	**0**		
Conducts at least one annual district-wide community physical activity event (e.g., fitness/wellness fair or 5K run/walk).	**3**	**2**	**1**	**0**		
**Section Totals**						

What would you say is the greatest strength of your school for helping children becoming more physically active?

What support or resources do you believe is needed to assist your students in becoming more physically active before, during, or after school?

PE Equipment Checklist - Secondary

Equipment and Materials	Current Number	Additional Wanted
**TEAM SPORTS (basketball, football, soccer, softball, volleyball, team handball, lacrosse, and field/floor hockey)**		
Basketballs		
Footballs		
Soccer balls		
Softball Equipment		
Volleyballs		
Team Handballs		
Lacrosse Sticks & Balls		
Field/floor hockey Equipment		
Other (please list):		
**OUTDOOR / ADVENTURE EDUCATION (adventure/ initiatives, backpacking, orienteering, geocaching)**		
Compasses		
GPS units		
Various equipment for adventure/cooperative initiatives		
Other (please list):		
**DANCE**		
Variable speed tape/CD player		
Music		
Other (please list):		

Equipment and Materials	Current Number	Additional Wanted
**INDIVIDAUL AND DAUL ACTIVITIES (gymnastics, archery, badminton, self-defense, golf, tennis, wrestling, track and field)**		
Rackets		
Golf clubs		
Bows/arrows		
Targets		
Hurdles		
High jump standards		
Discus		
Shot put		
Gymnastics equipment		
Other (please list):		
**RECREATIONAL ACTIVITIES (bowling, bocce, frisbee golf)**		
Bowling sets		
Boccee sets		
Frisbees		
Other (please list):		
**PHYSICAL FITNESS AND ASSESSMENT**		
Sit and reach box for measuring flexibility		
Stopwatches		
Modified Chin Up Bar and Standards		
Fitness Assessment Software Package		
Pedometers		
Pulse Bars/Heart Rate Monitors		
bioelectrical impedance machines		
Treadmills		
Ellipticals		
Stationary bikes		
Rowers		
Strength training equipment/dumbbells		
Step-aerobic boxes		
Jump ropes		
Other (please list):		

Equipment and Materials	Current Number	Additional Wanted
**ADAPTED/MODIFIED EQUIPMENT**		
**TECHNOLOGY**		
**OTHER (please list)**		

PE Equipment Checklist - Elementary

Equipment and Materials	Current Number	Additional Wanted
**EDUCATIONAL GAMES**		
Playground Balls		
Balls for Striking w/body		
Beach Balls		
Foam Balls		
Foam Soccer Balls		
Foam Footballs		
Fleece or Yarn Balls		
Balloons		
Squish Balls		
Foam Balls – Bounce-able		
Bean Bags		
Flying Disks		
Deck Rings		
Rag Balls		
Plastic Bottle Bats		
Soft-Bat		
Lollipop Paddles		
Scoops		
Youth Tennis Racquets		
Foam Blade Hockey Sticks		
w/Styrofoam Pucks		
Portable Gym Standards		
Nets for Standards		
Cones or Jug Markers		
Pinnies, Sashes, or Vests		
Scooter Boards w/Handles		
Spotmarkers		
Basketballs junior size		
Adjustable Basketball Goals		
Soccer Balls Sizes		
Lacrosse Sticks and Balls Soft headed sticks and hollow		
rubber balls		
Parachute		
Bowling Sets		
Over and Under Hurdles		
Volleyball Trainer Balls Lightweight volleyballs		
Other (please list):		

Equipment and Materials	Current Number	Additional Wanted
**EDUCATIONAL DANCE**		
Plastic Hoops		
Styrofoam Hoops Holders		
Rhythmic Equipment		
-drum w/mallet		
-lummi sticks		
Stretchy Material		
Nylon Juggling Scarves		
Tinikling Sticks/Boards and Jump Bands		
CD/Tape Player		
Cordless Microphone		
Sound System		
Other (please list):		
**EDUCATIONAL GYMNASTICS**		
Foam Vaulting Trapezoid		
Styrofoam Shapes Circles, triangles, ovals, wedges, etc.		
Mats		
2” thick, 100ILD foam 12-15 mats		
Landing Mats 4”-8” thick		
Incline Mats		
Balance Beams/Benches		
Balance Boards		
Jumping Boxes		
Foam Shapes of Varying Heights		
Trestles		
Sliding Boards to Connect to Trestles		
Connecting Ladder		
Hanging Climbing Ropes		
Jump Ropes		
Stretch Jump ropes		
Wands		
Other (please list):		

Equipment and Materials	Current Number	Additional Wanted
**PHYSICAL FITNESS AND ASSESSMENT**		
Sit and reach box for measuring flexibility		
Stopwatches		
Modified Chin Up Bar and Standards		
Fitness Assessment Software Package		
Pedometers		
Pulse Bars/Heart Rate Monitors		
bioelectrical impedance machines		
Treadmills		
Ellipticals		
Stationary bikes		
Rowers		
Strength training equipment/dumbbells		
Step-aerobic boxes		
Jump ropes		
Other (please list):		
**ADAPTED/MODIFIED EQUIPMENT**		
**TECHNOLOGY**		
**OTHER (please list)**		

Appendix C- PE Curriculum Content Worksheet

Instructions: Please review the list of PE units below. Mark an ‘x’ in corresponding boxes at which grade level the unit is offered at your school. Feel free to mark the “*Want more info”* box when appropriate**.**

**Activity**	**Grade Level**														
	PreK	K	1	2	3	4	5	6	7	8	9	10	11	12	Want more Info
Aerobics															
Angling/Casting															
Aquatics															
Archery															
Badminton															
Basketball															
Bicycling															
Bowling															
Cardio Kickboxing															
Cooperative Games															
Dance/Rhythms															
Fencing															
Field/Floor Hockey															
Fitness Testing															
Games/Relays															

Appendix C- PE Curriculum Content Worksheet

Activity	Grade Level													
	PreK	K	1	2	3	4	5	6	7	8	9	10	11	12	Want more Info
Golf															
Gymnastics															
Handball															
Inline Skating															
Lacrosse															
Jump Rope															
Locomotor Skill Development															
Manipulative Skill Development															
Non-Manipulative Skill Development															
Orienteering															
Physical Fitness/Conditioning															
Recreational Group Games															
Self-Defense															
Soccer															
Speedball															
Softball															
Table Tennis															

Appendix C- PE Curriculum Content Worksheet

**Activity**	**Grade Level**														
	PreK	K	1	2	3	4	5	6	7	8	9	10	11	12	Want more Info
Tennis															
Touch/Flag Football															
Track/Field															
Tumbling															
Volleyball															
Other (please list)															

Appendix D- Professional Development Activities Worksheet

Professional Development Activities Worksheet

Step 1: Please list the professional development experiences (workshops, classes, in-service presentations, webinars, etc.) you have participated in during the past 2 years.

**Workshop Title**	**Description**	**Month/Year**

Step 2: Select the professional development workshop topics that are of interest to you. If there are additional topics you would be interested in, please write in area below.

- ☐ Teaching Effectiveness
- ☐ FITNESSGRAM / ACTIVITYGRAM
- ☐ Standards-based Curriculum
- ☐ Assessment Strategies for K-12 PE
- ☐ NASPE Physical Best
- ☐ Using Technology in Physical Education
- ☐ Teaching Diverse Populations
- ☐ Teaching Students with Special Needs
- ☐ Integrating Strength & Conditioning
- ☐ Grant Writing
- ☐ State and National Standards
- ☐ Active Gaming in PE
- ☐ Integrating Health-related Fitness in PE
- ☐ Health Education
- ☐ New PE Units/Activities (e.g., tchoukball, mountain biking, archery, etc.)
- ☐ Comprehensive School Physical Activity Programming
- ☐ Active Academics – Taking PE and Physical Activity into the Classroom
- ☐ ____________________________________
- ☐ ____________________________________
- ☐ ____________________________________
- ☐ ____________________________________
- ☐ ____________________________________
- ☐ ____________________________________
- ☐ ____________________________________
- ☐ ____________________________________
- ☐ ____________________________________

Appendix E- Facilities and Play Space Audit Worksheet

Facilities and Play Space Audit Worksheet

**Facilities**	**Yes**	**No**	**Description and Use**
**OUTDOOR FACILITIES**			
**Outdoor Hard surface area**			
**Outdoor Dirt and/or turf area**			
**Playground for grades PreK-2**			
**Playground for grades 3-5**			
**Tennis courts**			
**Track and field facility**			
**Other (please list):**			
**INDOOR FACILITIES**			
**Gymnasium/instructional space**			
**Teacher office**			
**Locker rooms**			
**Classrooms**			

Modified from "Opportunity to Learn", a position statement of the National Association of Sport and Physical Education, 2004 and the Maryland State Department of Education Physical Education Facilities Guidelines, June 2011

Appendix E- Facilities and Play Space Audit Worksheet

Facilities	Yes	No	Description and Use
**Access to computer lab**			
**Bleachers**			
**Other (please list):**			
**OTHER**			
**Fitness center**			
**Fitness trails**			
**Weight room**			
**Aerobic/dance room**			
**Tumbling/gymnastics room**			
**Ropes course**			
**Equipment Storage**			
**Other (please list):**			

Modified from "Opportunity to Learn", a position statement of the National Association of Sport and Physical Education, 2004 and the Maryland State Department of Education Physical Education Facilities Guidelines, June 2011
